# Hospital Meetings

**Published:** 1906-03-17

**Authors:** 


### HOSPITAL MEETINGS.

### GREAT NORTHERN CENTRAL HOSPITAL.

In the absence of Sir John Dickson-Poynder, M.P., Chairman of the Committee of Management, the chair was taken by Dr. Burnet on the occasion of the annual meeting on March 8th.

The Chairman said that for some years past the work of the hospital had been steadily increasing, and last year was no exception to the rule. The average number of beds occupied was 147, and the average stay of each in-patient about 24 days, which meant a large number of patients passing through the wards during the year. Unfortunately the cost per bed was somewhat higher than last year. This was rendered inevitable by the fact that they had had to increase their nursing staff, which had got too low for the amount of work to be done. There had also been additional expenses connected with medical stores, bandages, and the surgical departments. But although the work had increased, and greater expenses been incurred, their income had rather fallen off. Annual subscriptions were less by £206 than in 1904; most of this was due to the death of Lord Cowper, who had subscribed £100 annually. It was not a healthy sign that the annual subscriptions should fall off. The Committee thought that the neighbourhood might do a great deal more in the way of subscriptions, however small. It was a very large district, and if a house-to-house collection could be organised, they might get a large sum of money, even in small amounts. The legacies last year were also small. The annual dinner had yielded a very good result. The Council of King Edward's Hospital Fund had given liberal donations, amounting in all to £3,100. But a good part of these sums went to capital account, and did not help them immediately in the matter of maintenance. During the last year they had lost the services of Miss Hull, the Matron, and Miss H. L. Pearse had been appointed in her place. At the present time a sub-committee was at work considering the question of the enormous number of out-patients, to see if by any means the number could not be reduced, and to guard against advantage being taken of the hospital by those who could afford to pay for treatment. The sub-committee would make their report later on.

The adoption of the report was moved by Mr. H. J. Tennant, M.P., who said it was obvious the hospital was increasing in efficiency, and in response to the demands of that great neighbourhood. The requirements of modern science had almost doubled of late years the duties of those who set themselves to meet the interests of the sick poor, and tried to keep abreast of the times; all this meant greater expendiure. Their position to-day was that while their usefulness had increased, the cost per bed had also increased. An almost automatically increasing expenditure was inevitable. The House and Financial Committees did their very best to keep down expenses, but it was almost impossible to keep them below the point at which they were now. For instance, an electrical department had had to be started. Those responsible for the management of hospitals felt they could not omit anything necessary for the most perfect treatment of the patients. They had under consideration schemes for the establishment of a children's ward, for a new arrangement for the housing of the nurses, and for starting a convalescent home.

The resolution was seconded by Mr. J. L. Smithett, who, in dealing with the present unsatisfactory state of the funds, said that Islington, comparatively speaking, gave scarcely anything towards the support of this noble institution, and seemed to take but little interest in philanthropic objects. The only subscriptions from the locality last year amounted to £600 and the donations to £200. Was it right, he asked, that a great and important place like Islington should have to go to the West End to get money to support its own charities? The amount raised in the neighbourhood by the festival dinner was £1,000, but there would be no dinner this year. There was a deficiency of £5,000 last year, and when they considered that they would have no funds from the dinner this year the Committee wondered if they might not be compelled to close a ward in the hospital. The Committee did not know where to turn for money. The Ladies' Association had done exceedingly well, and were willing to do more, but funds were urgently needed for the convalescent home, which everyone thought a necessity for the hospital. He appealed to all present to do something towards removing what was certainly a scandal upon Islington—namely, the present financial condition of their hospital.

Dr. Clifford Beale, in responding to a vote of thanks to the medical and surgical staff, said that it was during the last ten, or perhaps five, years that surgery had gone ahead at such a rate. At that hospital they wanted to keep the medical and surgical work abreast of the times, and did not want to be behind any other hospital. It was an expensive business, and if they were to keep up to date they must have more support. He concluded by referring with much regret to the recent death of Mr. W. R. H. Stewart, one of their surgeons.

The Chairman moved a vote of thanks to the Ladies' Association for the excellent work they had done, which was seconded by the Rev. W. H. Harwood, who remarked that the chief difficulty of the hospital was in creating local interest. There was much lack of local sympathy and of local desire to help institutions for the benefit of the poor of the neighbourhood. Coming from the North of England, he had especially noticed this absence of interest and sympathy.

Mr. J. L. Smithett, in moving that Miss Hull, the late Matron, should be elected an honorary life governor, bore handsome testimony to the good work she had done during her long connection with the hospital.

### ROYAL NATIONAL HOSPITAL FOR CONSUMPTION.

The annual general meeting of Governors was held at the London office in Craven Street on March 6.

The chair was taken by Lord Alverstone, the Lord Chief Justice, who, in moving the adoption of the annual report and balance-sheet, regretted the unavoidable absence of Lord Rosebery, their President. During the past year they had had 950 patients under treatment, 817 of whom had completed their stay there, 672 had gained weight, and a still larger number showed improvement from their stay in the hospital. Looking at the treatment in the hospital from a public point of view, he thought they had every reason to be proud of the results and to be satisfied with the good work the hospital was doing. He would at this stage like to pay a public tribute to the able assistance given by the medical staff, honorary and paid, and by the nurses in the care and attention of the patients. Considerable improvements had been effected during the past year, which those who had experience in such matters were of opinion would be to the advantage of the patients. A new dietary had been arranged for. There had also been new arrangements

